# Associations between circulating endostatin levels and vascular organ damage in systemic sclerosis and mixed connective tissue disease: an observational study

**DOI:** 10.1186/s13075-015-0756-5

**Published:** 2015-08-28

**Authors:** Silje Reiseter, Øyvind Molberg, Ragnar Gunnarsson, May Brit Lund, Trond Mogens Aalokken, Pål Aukrust, Thor Ueland, Torhild Garen, Cathrine Brunborg, Annika Michelsen, Aurelija Abraityte, Anna-Maria Hoffmann-Vold

**Affiliations:** Institute of Clinical Medicine, University of Oslo, 0318 Oslo, Norway; Department of Rheumatology, Oslo University Hospital Rikshospitalet, 0424 Oslo, Norway; Department of Respiratory Medicine, Oslo University Hospital Rikshospitalet, 0424 Oslo, Norway; Department of Radiology, Oslo University Hospital Rikshospitalet, 0424 Oslo, Norway; Department of Clinical Immunology and Infectious Diseases, Oslo University Hospital Rikshospitalet, 0424 Oslo, Norway; Research Institute of Clinical Medicine, Oslo University Hospital Rikshospitalet, 0424 Oslo, Norway; K. G. Jebsen Inflammation Research Centre, Institute of Clinical Medicine, University of Oslo, 0318, Oslo, Norway; K. G. Jebsen Thrombosis Research and Expertise Centre, The Arctic University of Norway, Langnes, 9037 Tromsø, Norway; Oslo Centre for Biostatistics and Epidemiology, Research Support Services, Oslo University Hospital, Ullevål, 0424 Oslo, Norway

## Abstract

**Introduction:**

Systemic sclerosis (SSc) and mixed connective tissue disease (MCTD) are chronic immune-mediated disorders complicated by vascular organ damage. The aim of this study was to examine the serum levels of the markers of neoangiogenesis: endostatin and vascular endothelial growth factor (VEGF), in our unselected cohorts of SSc and MCTD.

**Methods:**

Sera of SSc patients (N = 298) and MCTD patients (N = 162) from two longitudinal Norwegian cohorts were included. Blood donors were included as controls (N = 100). Circulating VEGF and endostatin were analyzed by enzyme immunoassay.

**Results:**

Mean endostatin levels were increased in SSc patients 93.7 (37) ng/ml (*P* < .001) and MCTD patients 83.2 (25) ng/ml (*P* < .001) compared to controls 65.1 (12) ng/ml. Median VEGF levels were elevated in SSc patients 209.0 (202) pg/ml compared to MCTD patients 181.3 (175) pg/ml (*P* = .017) and controls 150.0 (145) pg/ml (*P* < .001). Multivariable analysis of SSc subsets showed that pulmonary arterial hypertension (coefficient 15.7, 95 % CI: 2.2–29.2, *P* = .023) and scleroderma renal crisis (coefficient 77.6, 95 % CI: 59.3–100.0, *P* < .001) were associated with elevated endostatin levels. Multivariable analyses of MCTD subsets showed that digital ulcers were associated with elevated endostatin levels (coefficient 10.5, 95 % CI: 3.2–17.8, *P* = .005). The risk of death increased by 1.6 per SD endostatin increase (95 % CI: 1.2–2.1, *P* = .001) in the SSc cohort and by 1.6 per SD endostatin increase (95 % CI: 1.0–2.4, *P* = .041) in the MCTD cohort after adjustments to known risk factors.

**Conclusions:**

Endostatin levels were elevated in patients with SSc and MCTD, particularly SSc patients with pulmonary arterial hypertension and scleroderma renal crisis, and MCTD patients with digital ulcers. Elevated endostatin levels were also associated with increased all-cause mortality during follow-up in both groups of patients. We propose that endostatin might indicate the degree of vascular injury in SSc and MCTD patients.

**Electronic supplementary material:**

The online version of this article (doi:10.1186/s13075-015-0756-5) contains supplementary material, which is available to authorized users.

## Introduction

Systemic sclerosis (SSc) is a chronic multiorgan disease characterized by vasculopathy, progressive fibrosis of the skin and internal organs and distinct serum autoantibodies [1, 2]. The primary event in SSc is assumed to be vascular injury [3], which leads to clinical manifestations such as Raynaud’s phenomenon and digital ulcers [4]. Mortality is increased and mainly driven by pulmonary arterial hypertension (PAH) and pulmonary fibrosis [5]. Microangiopathy is thought to be responsible for the life-threatening organ involvement, such as PAH, scleroderma renal crisis (SRC), cardiomyopathy, gastric antrial vascular actasia [3] and possibly also pulmonary fibrosis [6].

Vascular injury has an impact in other connective tissue diseases (CTDs) and is particularly evident in mixed connective tissue disease (MCTD), a chronic immune-mediated disease associated with anti-U1-RNP autoantibodies and clinical features from SSc, systemic lupus erythematosus (SLE) and polymyositis (PM). MCTD appears to be genetically distinct from other CTDs [7], but there is an ongoing debate whether it should be categorized as a distinct disease, an overlap syndrome or an undifferentiated CTD [8]. Even though organ involvement in MCTD is more extensive than initially described, organ involvement is less severe than in SSc [9]. The vasculopathy in MCTD has been found to resemble the vasculopathy found in SSc [10] and it has been suggested that there is an association between pulmonary hypertension (PH) and anti-U1-RNP autoantibodies in SSc [11] and SLE [12]. Identifying SSc and MCTD patients at risk of developing serious vascular organ damage could improve patient outcome. Hence, there is a growing interest in markers that may predict vasculopathy [13].

In healthy tissue vascular injury causes hypoxia, which induces proteins in the vascular endothelial growth factor (VEGF) family. VEGF-A (usually referred to as VEGF) is released by a variety of cells including fibroblasts, macrophages, neutrophils, endothelial cells and T cells, and is involved in numerous steps of neoangiogenesis (13). Endostatin is the most potent inhibitor of VEGF-induced angiogenesis. It is a peptide derived from collagen XVIII, produced by fibroblasts and primarily found in the basement membranes of the skin and lungs [14], both of which are tissues involved in SSc and MCTD. Previous studies have shown increased serum levels of VEGF and endostatin in SSc [15] and MCTD [16], indicating an altered regulation of angiogenesis in these diseases. However, the previous studies of VEGF and endostatin in SSc and MCTD were performed in small-scale cohorts, and the correlation to clinical parameters has been somewhat discrepant [17–19] and some have been contradictive [17, 20]. An additional table shows previous data in more detail (see Additional file 1) [16–24].

The aim of this study was to assess the serum levels of endostatin and VEGF in two large, well-characterized and largely unselected longitudinal CTD cohorts; the Oslo University Hospital (OUH) SSc cohort [25, 26] and the Norwegian nationwide MCTD cohort [27–29]. Serum levels of endostatin and VEGF of SSc and MCTD patients were compared with controls. Our basic hypothesis was that VEGF and endostatin were associated with vasculopathy-related features like digital ulcers, PAH, SRC and possibly also pulmonary fibrosis, in both diseases. Hence, we wanted to explore the associations of these clinical parameters and all-cause mortality with endostatin and VEGF levels.

## Methods

### Study cohorts

Sera of SSc patients (N = 298) from the previously described Oslo University Hospital (OUH) SSc cohort were assessed [25, 30, 31]. The OUH SSc cohort is an observational prospective study cohort which includes all consenting SSc patients seen at OUH since 2008. Patients included in the cohort have annual follow-up visits at OUH where clinical parameters, pulmonary function tests (PFTs), lung high-resolution computed tomography (HRCT), echocardiography, and right-sided heart catheterization (RHC) data are systematically recorded and stored in the Norwegian Systemic Connective Tissue Disease and Vasculitides Registry (NOSVAR) [26]. Serum samples are taken at inclusion and stored in the NOSVAR biobank. All SSc patients included in this study fulfilled the 2013 European League Against Rheumatism/American College of Rheumatology (EULAR/ACR) classification criteria for SSc [25, 32].

Sera of MCTD patients (N = 162) from the previously described unselected Norwegian nationwide MCTD cohort [7, 27–29] (n = 135) and NOSVAR (n = 27) were similarly assessed. The Norwegian nationwide MCTD cohort recruited patients from Departments of Rheumatology from 2005 to 2008, while NOSVAR recruited patients from OUH from 2008 to 2012. Inclusion criteria were age 18 and fulfillment of at least one of the three criteria sets of MCTD, the modified Sharp’s criteria [33], the criteria of Alarcón-Segovia or Kasukawa [9], and the exclusion of another CTD [29]. Consenting blood donors from the OUH blood bank were included as controls (N = 100).

### Clinical parameters

SSc patients were categorized as diffuse cutaneous SSc (dcSSc) or limited cutaneous SSc (lcSSc) [34]. Disease onset was defined as the onset of the first non-Raynaud’s symptom. Clinical parameters recorded included percentage of predicted full vital capacity (FVC) and percentage of predicted diffusing capacity of the lung for carbon monoxide (DLCO), pulmonary fibrosis (by HRCT: see below), digital ulcers, SRC and PH segregated into two well-defined groups; PAH and PH due to lung disease. In the MCTD cohort, clinical parameters included percentage of predicted FVC and DLCO, pulmonary fibrosis, digital ulcers, PAH and PH due to lung disease. Digital ulcers were scored positive if ulcers were present at least once during the disease course. Precapillary PH was defined according to the updated European Society of Cardiology (ESC) criteria by mean pulmonary artery pressure (mPAP) ≥25 mm HG and pulmonary wedge pressure ≤15 mm HG by RHC at rest [35]. Patients in our SSc cohort have an annual clinical follow-up at OUS, they are referred to RHC when it is clinically indicated based on physical examination, PFT results, 6-minute walk tests and echocardiogram results. Echocardiogram results were available in 294 patients (99 %). RHC was performed in 96 patients (32 %). In the analyses we included patients classified as having pulmonary arterial hypertension (PAH). PAH is characterized by the presence of precapillary PH in the absence of other causes of precapillary PH such as PH due to lung diseases, chronic thromboembolic PH, or other rare diseases [35]. Vital status at the end of the study was obtained from the National Population Register of Norway.

### High-resolution computed tomography (HRCT) analysis of the lungs and pulmonary function tests (PFTs)

The presence of fibrosis was evaluated according to the CT criteria of interstitial lung disease recommended by The Nomenclature Committee of the Fleischner Society [36]. HRCT was obtained by inclusion in both cohorts, and reviewed on a Picture Archiving and Communication System (PACS) screen independently and in random order by two experienced readers. HRCT were available in 252 of 298 patients (85 %) in the SSc cohort and in 148 of 162 patients (91 %) in the MCTD cohort. Pulmonary function tests were performed according to established guidelines [37, 38].

### Blood samples

Blood samples were centrifuged at room temperature after 30 minutes and serum aliquots were stored at −70 °C until assayed. Circulating VEGF and endostatin were analyzed by enzyme immunoassay (R&D Systems, Stillwater, MN, USA). The detection limit for endostatin was 80 pg/ml and intra- and interassay coefficients <10 %.

### Statistical analyses

Serum levels of endostatin were compared by means (M) and standard deviation (SD) in all groups. Statistical differences between MCTD, SSc and controls were analyzed by one-way ANOVA. Post hoc comparisons were performed using Tukey’s test. Serum levels of VEGF did not have a normal distribution and were presented as median (Mdn) and interquartile range (IQR). Comparisons between the three groups were analyzed by Kruskal-Wallis test. Estimations of the effects of various clinical manifestations on serum endostatin levels were performed by linear regression analyses. We included clinical parameters with evident vasculopathy; digital ulcers, PAH and SRC. Since it has been proposed that vasculopathy is involved in pulmonary fibrosis this parameter was included together with the accompanying lung parameters; percentage of predicted FVC and percentage of predicted DLCO. Known risk factors for SRC and PAH was also included in the model. In the linear regression analyses we included patients with established PAH (N = 24) and SRC (N = 11) at the year of serum sampling. Univariable and multivariable logistic regression analyses were performed to explore the predictive value of endostatin. Patients who developed PAH and SRC the same year as serum sampling or later were included in the logistic regression analyses (N = 16 and N = 6, respectively). An additional graph shows when patients were diagnosed with PAH and SRC in relation to serum sampling (Additional file 2). Variables that were significant in univariable analyses were included in multivariable analysis. Using a manual backward elimination procedure, variables at a significant level of *P* < .25 in the univariable analyses were considered a candidate for the multivariable model in conjunction with age and gender. The association was quantified by the odds ratio (OR) with its 95 % confidence interval (CI). With regard to follow-up time, participants were followed from the date of inclusion in the cohort until death or end of follow-up on 31 October 2014. Kaplan-Meier survival curves were used to determine difference in survival between tertiles of endostatin levels and were estimated by the log-rank test. A multivariable Cox regression model was performed to control for multiple confounders. The proportional hazard assumptions were tested by plotting the logarithm of the integrated hazards (log–log survival plots). The effects were quantified by hazard ratio (HR) with its 95 % CI. Known risk factors were included in the multivariable logistic [39, 40] and Cox regression analyses [30, 41]. The significance level was set at *P* ≤ .05. Data extraction and analyses were conducted using SPSS version 22 (IBM SPSS, Armonk, NY, USA) and STATA version 22 (StataCorp, College Station, TX, USA).

### Ethics

The study was approved by the Norwegian Regional Committee for Medical and Health Research Ethics and conducted in accordance with the guidelines of the Helsinki II declaration. All patients have given informed written consent to participate in the study.

## Results

### Serum endostatin and VEGF levels in the study cohorts

**Fig. 1:**
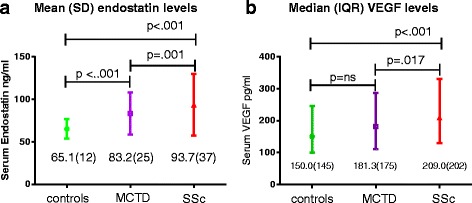
**a**-**b** Endostatin and vascular endothelial growth factor (VEGF) serum levels in systemic sclerosis (SSc), mixed connective tissue disease (MCTD) and controls

Circulating endostatin and VEGF levels were assessed in the OUH SSc cohort (N = 298) and in the Norwegian MCTD cohort (N = 162) (Table 1). Mean (SD) serum endostatin was higher in SSc 93.7 (37) ng/ml than MCTD 83.2 (25) ng/ml (*P* = .001) and controls 65.1 (12) ng/ml (*P* < .001). Mean serum endostatin was also higher in MCTD compared to controls (*P* < .001) (Fig. 1a). The SSc patients had higher median VEGF 209.0 (IQR 202) pg/ml than both MCTD 181.3 (175) pg/ml (*P* = .017) and controls 150.4 (145) pg/ml (*P* < .001). VEGF levels did not differ between MCTD and controls (Fig. 1b).

**Table 1:** Demographics and clinical parameters of the MCTD and SSc cohorts

	SSc	MCTD
Patients, N	298	162
Females, N (%)	243 (82)	128 (79)
Diffuse cutaneous SSc, N (%)	78 (26)	N/A
Age at diagnosis, years, M (SD)	48.3 (15.4)	35.4 (15.7)
Age at blood sampling, M (SD)	56.0 (13.8)	44.7 (14.9)
Disease duration at sampling, years, Mdn (IQR)	4.0 (9)	7.0 (7)
Deceased, N (%)	58 (20)	14 (9)
Observation time, months, M (SD)	55.0 (28.7)	98.8 (27.4)
Classification criteria:		
ACR/EULAR 2013 SSc, N (%)	298 (100)	N/A
Alarcon, N (%)	N/A	143 (88)
Sharp, N (%)	N/A	151 (93)
Kasukawa, N (%)	N/A	142 (88)
Pulmonary fibrosis at sampling, N (%)	103/252 (40)	52/148 (35)
% of predicted FVC, N (%)	297 (100)	146 (90)
% of predicted FVC, M (SD)	94,7 (20,5)	92.1 (18.5)
% of predicted DLCO, N (%)	295 (99)	142 (88)
% of predicted DLCO, M (SD)	68.2 (21.7)	73.8 (16.4)
Digital ulcers, N (%)	132/278 (44)	50/155 (32)
Precapillary pulmonary hypertension, N (%)	44 (15)	3 (.05)
Pulmonary arterial hypertension, N (%)	32 (10.7)	2 (.04)
Scleroderma renal crisis, N (%)	12 (4)	0 (0)

*MCTD* mixed connective tissue disease, *SSc* systemic sclerosis, *N* numbers, *N/A* not applicable, *M* mean, *SD* standard deviation, *Mdn* median, *IQR* interquartile range, *FVC* forced vital capacity, *DLCO* diffusing capacity of the lungs for carbon monoxide

### Association between clinical parameters and serum endostatin and VEGF in the SSc cohort

**Fig. 2:**
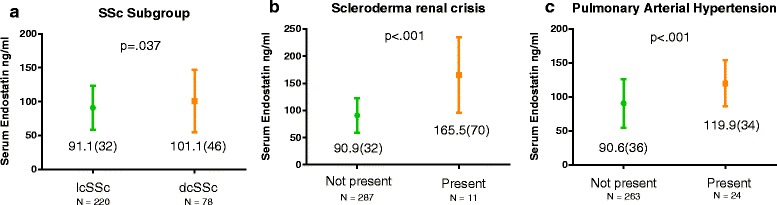
**a**, **b** and **c** Mean endostatin levels in different systemic sclerosis (SSc) subsets

In univariable analyses dcSSc (Fig. 2a), SRC (Fig. 2b) and PAH (Fig. 2c) were associated with elevated endostatin levels, while percentage of predicted DLCO was negatively associated with endostatin levels (Table 2). In the multiple linear regression analysis, PAH and SRC were significantly associated with elevated endostatin levels (Table 2). The strongest effect was SRC with a mean difference of 77.6 ng/ml in endostatin levels between patients with and without SRC.

**Fig. 3:**
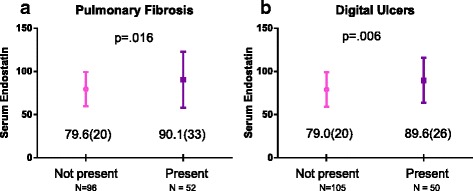
**a** and **b** Mean endostatin levels and clinical parameters in mixed connective tissue disease (MCTD)

**Table 2:** Association between clinical parameters and circulating endostatin in the SSc cohort

Clinical manifestations	Univariable			Multivariable^a^		
	Regression coefficient	95 % CI	*P* value	Regression coefficient	95 % CI	*P* value
% of predicted FVC	−.14	−.34, −.07	.192			
% of predicted DLCO	−.33	−.52, −.15	.001	−.18	−.35, −.002	.048
dcSSc vs. lcSSc	10.0	.6, 19.5	.037			
Pulmonary arterial hypertension	29.3	14.4, 44.2	< .001	15.7	2.2, 29.2	.023
Scleroderma renal crisis	74.6	54.1, 95.0	< .001	77.6	59.3, 100.0	< .001

*SSc* systemic sclerosis, *CI* confidence interval, *FVC* forced vital capacity, *DLCO* diffusing capacity of the lungs for carbon monoxide, *dsSSc* diffuse cutaneous SSc, *lcSSc* limited cutaneous SSc

^a^Variables in the final multivariable regression model: percentage of predicted DLCO, pulmonary arterial hypertension, scleroderma renal crisis, age and gender

An inverse association was found between percentage of predicted DLCO and elevated levels of VEGF pg/ml (coefficient - .14, 95 % CI −2.5, −.3, *P* = .013). No other associations were found between serum VEGF and clinical parameters in the SSc cohort.

### Association between clinical parameters and serum endostatin and VEGF in the MCTD cohort

Pulmonary fibrosis (Fig. 3a) and digital ulcers (Fig 3b) were associated with high endostatin serum levels, while a negative association was found with percentage of predicted FVC and DLCO in the univariable analyses (Table 3). In the multivariable linear regression analyses digital ulcers remained significant, indicating a mean difference of 10.5 ng/ml in endostatin levels in patients with and without digital ulcers. No significant associations were found between clinical parameters and serum VEGF in the MCTD cohort.

**Table 3:** Association between clinical parameters and circulating endostatin in the MCTD cohort

Clinical manifestations	Univariable			Multivariable^a^		
	Regression coefficient	95 % CI	*P* value	Regression coefficient	95 % CI	*P* value
Pulmonary fibrosis	10.5	1.9, 19.1	.017			
% of predicted FVC	− .33	−.55, −.12	.002			
% of predicted DLCO	−.40	−.64, −.16	.001			
Digital ulcers	10.7	3.2, 18.2	.006	10.5	3.2, 17.8	.005

*MCTD* mixed connective tissue disease, *FVC* forced vital capacity, *DLCO* diffusing capacity of the lungs for carbon monoxide

^a^Variables in the final multivariable regression model: digital ulcers, age and gender

### Predictive value of endostatin in SSc

Logistic regression was performed to explore the predictive value of endostatin. When assessing all SSc patients who developed PAH after or within the year of endostatin samples were taken, no significant association was found. However, when including patients that developed PAH within the first 2 years after blood sampling we found for each one SD increase in endostatin levels the odds of developing PAH increased with 70 % (OR = 1.7, 95 % CI: 1.2–2.4, *P* = .005) (see Additional file 3). The predictive value of endostatin for PAH development was not significant in the multivariable analysis. We then assessed patients who developed SRC after blood sample. All six cases were diagnosed within 2 years of blood sampling. A one SD increase in endostatin level in SSc was associated with a 3.2-fold higher odds (95 % CI: 1.8–5.7, *P* < .001) of developing SRC. Endostatin was identified as the only predictor of SRC after assessing candidates for multivariable analyses [40] including age, gender, disease duration and dcSSc (see Additional file 3). Analyzing the predictive value of endostatin in the MCTD cohort was not applicable.

### All-cause mortality and endostatin levels in the SSc cohort

**Fig. 4:**
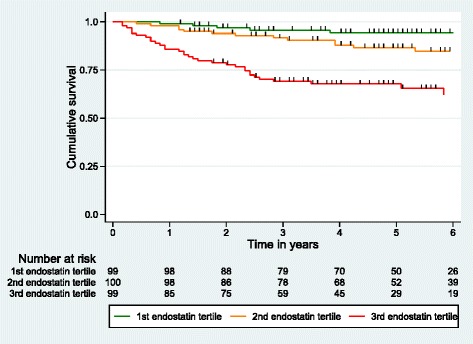
Kaplan-Meier curve for tertiles of endostatin

During 5 years of follow-up 48 patients died and during 10 years of follow-up 58 SSc patients died. SSc patients were divided in tertiles of endostatin levels. The 5-year cumulative survival rate was 94 % (95 % CI: 87–98 %) in the first tertile, 87 % (95 % CI: 77–92 %) in the second tertile and 68 % (95 % CI: 57–76 %). The 10-year cumulative survival rate was 94 % (95 % CI: 90–99 %) in the first tertile, 64 % (95 % CI: 39–86 %) in the second tertile and 28 % (95 % CI: 0–56 %) in the third tertile (log rank *P* < .001) (Fig. 4). The risk of death increased by 1.6 per SD endostatin in multivariable Cox regression analysis when adjusting for the confounding effects of age, gender, disease duration, dcSSc, pulmonary fibrosis, PH and SRC (95 % CI: 1.2–2.1 %, P = .001).

### All-cause mortality and endostatin levels in the MCTD cohort

Similar analyses were performed in the MCTD group. During 10 years of follow-up 14 MCTD patients died and the 10-year cumulative survival rate was 92 % (95 % CI: 85–99 % in the first tertile, 91 % (95 % CI: 77–100 %) in the second tertile and 77 % (95 % CI: 62–92 % in the third tertile. Following multivariable Cox regression a one SD increase in endostatin level increased the risk of death by 1.6 (95 % CI: 1.0–2.4 %, *P* = .041) when adjusting for the confounding effects of age, gender, disease duration, pulmonary fibrosis and PH.

## Discussion

Identifying SSc and MCTD patients at risk of developing serious vascular organ damage could improve patient outcome. The main findings of this study were that increased circulating endostatin, but not VEGF was independently associated with PAH and SRC in SSc patients and with digital ulcers in MCTD patients. Survival analysis showed higher all-cause mortality in both SSc and MCTD patients with increased endostatin levels. Endostatin has been found to be increased in MCTD and SSc compared to controls in previous small-scale studies [16, 18, 19, 23, 24]. The present study extends these previous findings in a much larger sample size and shows a strong association with severe vascular organ damage and mortality during long-term follow-up.

We found higher levels of endostatin in SSc than in MCTD, possibly reflecting that the inhibition of angiogenesis is greater in SSc than in MCTD [14]. In line with other studies [17–19, 23], we found serum VEGF levels to be elevated in SSc compared to controls. Elevated VEGF levels in blood and skin of SSc patients have previously been suggested to contribute to the chaotic capillary morphology seen in these patients [42]. In contrast to other studies [16, 17, 22], we did not find associations between VEGF levels and clinical parameters in the SSc or MCTD cohorts. Importantly, our findings support the study by Hummers et al. showing increased levels of endostatin and not VEGF in SSc patients with PH [23].

The mechanisms behind PAH and SRC development in SSc are not fully understood. The pathology in SSc PAH is described as an obliterative vasculopathy with intimal proliferation, medial hyperplasia, and adventitial fibrosis in the small pulmonary arterioles [43], while thrombotic microangiopathic vasculopathy has been observed in SRC [44]. The current endostatin data supports the hypothesis that dysregulated angiogenesis may play a role in both PAH and SRC. Moreover, recent studies have suggested that endostatin may influence the regulation of matrix metalloproteinases [45], which could contribute to vascular remodeling in the SSc target organs [46].

Previous data from a small MCTD cohort with cases selected from referral centers showed that the patient subsets with acrosclerosis and PH had high circulating VEGF, but endostatin were the same levels as controls [16]. In the present larger and population-based MCTD cohort we found an association between digital ulcers and elevated endostatin levels, implying that the level of endostatin might reflect the severity of vasculopathy in MCTD. In the unselected Norwegian MCTD cohort, PAH was less frequent (two cases in total) than other studies have shown [47, 48]. Due to the low number we could not perform analyses involving endostatin and PAH in the MCTD cohort.

For clinical purposes, we found it of interest to explore the potential predictive value of endostatin. These analyses showed that increasing endostatin levels predicted PAH development within 2 years in SSc patients in the univariable analysis, but not in multivariable analysis where age and percentage of predicted DLCO had stronger predictive value. Since both are well-known risk factors for PAH in SSc [39] there is still a possible role for endostatin in predicting PAH, but this needs to be further investigated in cohorts with larger numbers of PAH cases. We found endostatin to be the only predictor of SRC, however due to low number of SRC events (six in total), results must be carefully interpreted.

The present study is, to our knowledge, the first to show an association between endostatin and all-cause mortality in SSc and MCTD. Previous studies have reported elevated endostatin to be associated with increased risk of death in the elderly [49] and a predictor of all-cause mortality in patients with chronic heart failure of ischemic origin and poor renal function [50].

A major strength of this study is the large number of included patients with MCTD and SSc, and the comparison of results between two diseases with partly overlapping clinical features from the two cohorts. There was no loss to follow-up. The cohorts are largely unselected and they have a longitudinal study design that consists of comprehensive clinical data. This gave us the opportunity to assess a number of relevant parameters in the multivariable analyses. In addition to the clinical parameters shown in Tables 2 and 3, we also assessed age, gender, disease duration, digital ulcers and pulmonary fibrosis in both cohorts, and sclerodactyly and puffy hands in the MCTD cohort only.

A limitation of this study was not distinguishing the proangiogenic and antiangiogenic isoforms of VEGF [51]. Unfortunately, we were not able to assess the associations of VEGF and endostatin to the clinical vasculopathy features cardiomyopathy and gastric antrial vascular actasia due to missing data in our SSc cohort. We were not able to compare endostatin to known cardiovascular risk factors in our study and we were not able, due to missing data, to adjust for pro-brain natriuretic peptide serum levels, estimation of glomerular filtration rate levels or anti-RNA polymerase antibodies. For the parameters SRC in SSc and deaths in MCTD the numbers were low, weakening the impact of these findings. Finally, the endostatin and VEGF measurements were performed cross-sectionally at different disease durations.

## Conclusions

In this study, performed in largely unselected patient cohorts, we demonstrated that endostatin levels are elevated in SSc and MCTD patients, and associated with SRC and PAH in SSc patients and digital ulcers in MCTD patients. High endostatin levels were also associated with increased all-cause mortality during follow-up in both groups of patients. Taken together our data further underscore the role of dysregulated angiogenesis in SSc and MCTD and suggest that endostatin could reflect the degree of vasculopathy in these disorders. Further studies are warranted to evaluate the potential role of circulating endostatin as a marker for serious vascular organ damage in SSc and MCTD patients.

## Additional files

Additional file 1:
**Previous studies of endostatin and/or vascular endothelial growth factor in SSc and MCTD.** Listing of previous studies of endostatin and/or vascular endothelial growth factor levels in SSc and/or MCTD including methods and main results. (PDF 241 kb) Additional file 2:
**Diagnoses of pulmonary arterial hypertension and scleroderma renal crisis in relation to serum sampling time.** A graph showing time of diagnosis of pulmonary arterial hypertension and scleroderma renal crisis in relation to time of serum sampling. (PDF 122 kb) Additional file 3:
**Results of logistic regression analyses.** Known risk factors and endostatin in predicting pulmonary arterial hypertension and scleroderma renal crisis. (PDF 282 kb)

## Abbreviations

CI: confidence interval
CTD: connective tissue disease
dcSSc: diffuse cutaneous SSc
DLCO: diffusing capacity of the lungs for carbon monoxide
FVC: forced vital capacity
HRCT: high-resolution computed tomography
IQR: interquartile range
lcSSc: limited cutaneous SSc
M: means
MCTD: mixed connective tissue disease
Mdn: median
NOSVAR: Norwegian Systemic Tissue Disease and Vasculitides Registry
OR: odds ratio
OUH: Oslo University Hospital
PAH: pulmonary arterial hypertension
PFT: pulmonary function test
PH: pulmonary hypertension
RHC: right-sided heart catheterization
SD: standard deviation
SLE: systemic lupus erythematosus
SRC: scleroderma renal crisis
SSc: systemic sclerosis
VEGF: vascular endothelial growth factor
