# Supplementary material for: Associations between circulating endostatin levels and vascular organ damage in systemic sclerosis and mixed connective tissue disease: an observational study
Source: Arthritis Res Ther. 2015 Aug 28;17(1):231. doi: 10.1186/s13075-015-0756-5 (PMC4551562; doi:10.1186/s13075-015-0756-5)
Supplement: Additional file 1: — Previous studies of endostatin and/or vascular endothelial growth factor in SSc and MCTD. Listing of previous studies of endostatin and/or vascular endothelial growth factor levels in SSc and/or MCTD including methods and main results. (PDF 241 kb) [file 13075_2015_756_MOESM1_ESM.pdf]

Previous studies of endostatin and/or vascular endothelial growth factor in SSc and/or MCTD

Study	Participants	Methods	Main results
Hebbar 2000 (16)	50 SSc<br>30 Ctrls	Student t-test and Spearmann Rank Test	**Serum Endostatin:** SSc>Ctrls. SSC with>without digital ulcers. SSc abnormalities on chest x-ray present>not present.
Farouk 2013 (17)	25 SSc<br>20 Ctrls	Unpaired t test, ANOVA and univariable Linear regression	**Serum VEGF:** SSc>Ctrls. Less than 3 years disease duration > More than 3 years disease duration. Without>with digital ischemic manifestations.<br>**Serum Endostatin:** SSc>Ctrls. More than 3 years disease duration>Less than 3 years disease duration. With>without digital ischemic manifestations. With> without restricted PFT's. Correlated to increasing histopathological thickness score
Choi 2003 (18)	48 SSc<br>30 Ctrls	Mann Whitney U test and Spearman's rank correlation	**Serum VEGF:** SSc>Ctrls. Diffuse>Limited cutaneous SSc. Correlated to Rodnan Skin Score. Negatively correlated to number of capillary loops on nailfold capillaroscopy.
Dziankowska 2005 (19)	34 SSc<br>20 Ctrls	Mann-Whitney U test, Cochrane Cox Two independent sample test and Fisher precisely test	**Serum VEGF:** Ctrls >SSc<br>**Serum Endostatin:** SSc>Ctrls. With > without cardiovascular abnormalities*
Distler 2011 (20)	38 MCTD<br>66 Ctrls<br>40 SSc	Mann-Whitney U test, Pearson's and Spearman's rank correlation test	**Serum VEGF**: MCTD>Ctrls. SSc=MCTD, MCTD with > without acrosclerosis. MCTD with > without myositis**. MCTD with > without PH***<br>**Serum Endostatin:** MCTD>Ctrls. SSc=Ctrls.
De Santis 2012 (21)	55 SSc<br>17 Ctrls	Mann–Whitney's test, Wilcoxon's rank sum test and Spearman's rank correlation	**BALF VEGF:** Ctrls > SSc. SSc without > with alvolitis****. Inverse correlation to alveolar score.
Distler 2002 (22)	43 SSc<br>21 Ctrls	The Kruskal–Wallis test, the Mann–Whitney test and the Spearman's rank test	**Serum VEGF:** SSc > Ctrls, Diffuse > Limited, Positive > Negative Anti-Scl-70 Without > With Digital Ulcers,<br>**Serum Endostatin:** SSc = Ctrls, Giant capillaries on capillaroscopy: Present > Not present
Hummers 2009 (23)	113 SSc<br>27 Ctrls	Mann-Whitney U-test, Spearman's rank correlation, simple and multivariate linear regression and ordered logistic regression	**Serum VEGF:** SSc > Ctrls<br>**Serum Endostatin:** SSc > Ctrls, Positiv correlation to estimated right ventricular systolic pressure (eRVSP) measured by ECHO. Pulmonary Hypertension present > not present<br>Negative correlation to FVC
Dziankowska 2006 (24)	28 SSc<br>20 Ctrls	The Mann-Whitney U test and the Spearman test	**Serum Endostatin:** SSc > Ctrls, SSC with ILD***** > Ctrls<br>**Serum VEGF – Endostatin Ratio:** SSc < Ctrls<br>**Serum VEGF:** SSc = Ctrls

Systemic Sclerosis (SSc), Mixed Connective Tissue disease (MCTD), Controls (Ctrls), Vascular Endothelial Growth Factor (VEGF)

* Tendency to tachyarhythmias, lower potentials of different parameters describing variability of cardiac rhythm or conduction system abnormalities, ventricular arrhythmia, silent ischaemic episodes, diastolic left ventricular disturbances,and valvular lesions.

** Diagnosed when at least twofold elevated serum CK levels and characteristic electromyographic changes were detected or when myositis was proven histologically .

*** Diagnosed by right heart catheterization.

**** Diagnosed by BALF when the percentage of neutrophils was 3% and/or eosinophils 2%. Alveolar score on HRCT: ground glass opacity on a scale of 0–5 in the three lobes of both lungs.

***** Considered present if (a) DLCO corrected< 80% of predicted plus either b) bilateral reticulo-nodular shadowing predominantly involving the lower lobes of the chest X-ray and or high resolution computed tomography , (c) bilateral inspiratory crackles on clinical chest examination.
