# Supplementary figures and images for: Associations between circulating endostatin levels and vascular organ damage in systemic sclerosis and mixed connective tissue disease: an observational study
Source: Arthritis Res Ther. 2015 Aug 28;17(1):231. doi: 10.1186/s13075-015-0756-5 (PMC4551562; doi:10.1186/s13075-015-0756-5)

## Precapillary Arterial Hypertension

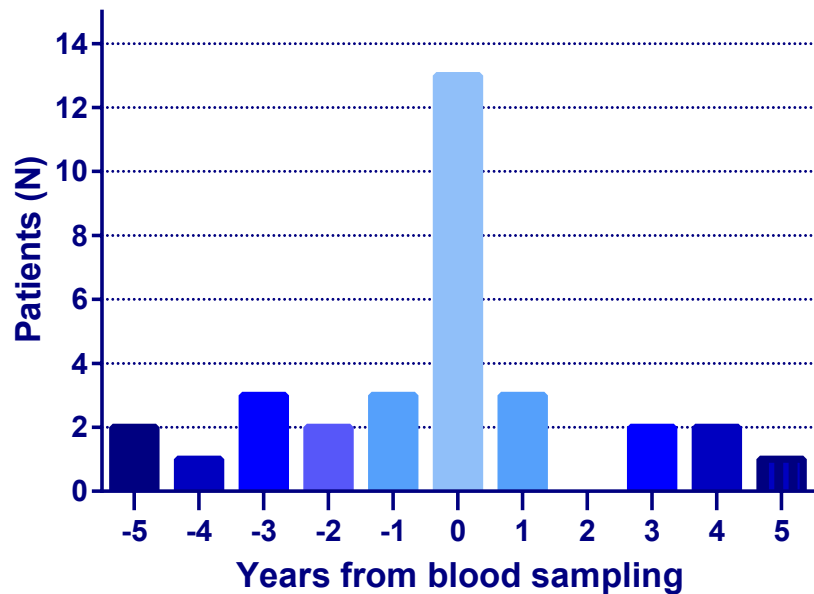

## Scleroderma renal crisis

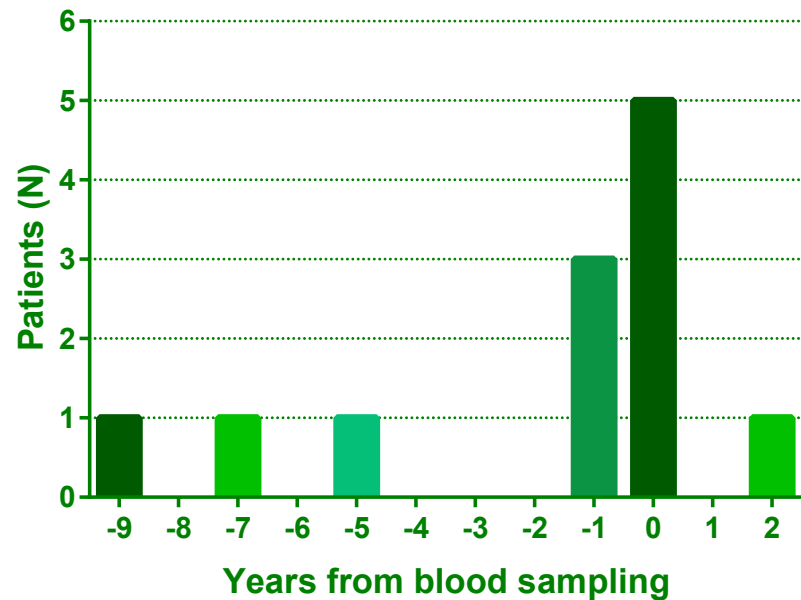

Supplement: Additional file 2: — Diagnoses of pulmonary arterial hypertension and scleroderma renal crisis in relation to serum sampling time. A graph showing time of diagnosis of pulmonary arterial hypertension and scleroderma renal crisis in relation to time of serum sampling. (PDF 122 kb) [file 13075_2015_756_MOESM2_ESM.pdf]
