# Supplementary material for: Associations between circulating endostatin levels and vascular organ damage in systemic sclerosis and mixed connective tissue disease: an observational study
Source: Arthritis Res Ther. 2015 Aug 28;17(1):231. doi: 10.1186/s13075-015-0756-5 (PMC4551562; doi:10.1186/s13075-015-0756-5)
Supplement: Additional file 3: — Results of logistic regression analyses. Known risk factors and endostatin in predicting pulmonary arterial hypertension and scleroderma renal crisis. (PDF 282 kb) [file 13075_2015_756_MOESM3_ESM.pdf]

Results of logistic regression analyses

Pulmonary Arterial Hypertension (within 2 years from serum sampling)	Univariable			Multivariable*		
	OR	95% CI	p-value	OR	95% CI	p-value
1 SD change in endostatin (37 ng/ml)	1.7	1.2– 2.4	P=.005			N.S
Gender	2.2	0.7-6.7	0.115	2.9	0.8-11.0	0.119
Age	1.1	1.1-1.2	P<.001	1.1	1.1-1,2	0.001
DLCO %	0.9	0.9-1.0	P<.001	0.9	0.9-1.0	0.004
**Scleroderma Renal Crisis (all within 2 years from serum sampling)**	**Univariable**			**Multivariable****		
	OR	95% CI	p-value	OR	95% CI	p-value
1 SD change in endostatin (SD=37 ng/ml)	3.2	1.8– 5.7	P<.001	N. A.		

* Digital ulcers and disease duration at sampling had P>.25 and were therefor not included in the multivariable model
**Disease duration at sample, age, gender and dcSSc had P>.25. Only 1-SD increase in endostatin was found to be statistically significant in predicting Scleroderma Renal Crisis
N/A : not applicable, N.S : Not significant
